# A Single Bout of High-Intensity Exercise Session Affects Salivary Biomarkers of Creatine Metabolism in Healthy Young Men and Women

**DOI:** 10.1080/15502783.2025.2533660

**Published:** 2025-07-18

**Authors:** Bogdan Andjelic, Nikola Todorovic, Jovana Panic, Milan Vranes, Valdemar Stajer, Sergej M. Ostojic

**Affiliations:** a Applied Bioenergetics Lab, Faculty of Sport and Physical Education, University of Novi Sad, Novi Sad, Serbia; b Sport and Exercise Science Research Unit, University of Palermo, Palermo, Italy; c Faculty of Sciences, University of Novi Sad, Novi Sad, Serbia; d Faculty of Health Sciences, University of Pécs, Pécs, Hungary; e Department of Nutrition and Public Health, University of Agder, Kristiansand, Norway

**Keywords:** Exercise, creatine, saliva, biomarkers, metabolism, high-intensity interval training

## Abstract

**Background:**

Energy requirements during high-intensity exercise rapidly escalate. Phosphocreatine, being the major compound utilized to rapidly replenish the ATP levels, essentially relies on creatine stores which may experience fluctuations in its utilization, transport or release across various bodily fluids. So far, no studies have assessed whether exercise affects salivary biomarkers of creatine metabolism, or whether these metabolites in saliva mirror those found in the serum.

**Method:**

Sixteen healthy adults volunteered in this open-label crossover pilot study. All participants were subjected to the running-to-exhaustion treadmill protocol, starting with a 3-minute warm-up at 5 km/h, progressively increasing the workload by 1.5 km/h every minute. Fasted blood samples were collected from the antecubital vein and unstimulated whole saliva was collected from beneath the tongue ~ 30 minutes before and immediately after intervention. All samples underwent analysis for creatine and creatinine using a modified liquid chromatography–tandem mass spectrometry. Differences were compared using paired two-tailed t-test and Pearson’s correlation was used to evaluate the linear relationship.

**Results:**

Fifteen participants (23.9 ± 2.9 years, eight females) were analyzed for the study outcomes. In contrast to serum, salivary creatine and creatinine levels significantly elevated pre- to post-exercise (17.5 ± 14.2 µmol/L to 43.6 ± 30.4 µmol/L and 11.3 ± 5.8 µmol/L to 17.0 ± 9.3 µmol/L, *P* < 0.001, *P* = 0.04). Salivary creatine and creatinine metabolism showed a non-significant correlation with the corresponding serum biomarkers at baseline (*r* = 0.18, *P* = 0.51, and *r* < 0.01, *P* = 0.99) and after the exercise (*r* = 0.33, *P* = 0.21 and *r* = 0.40, *P* = 0.13).

**Conclusion:**

Our preliminary findings suggest a potential difference in the exercise-induced dynamics of creatine metabolism across the two bodily fluids, warranting further research into using saliva as simple and non-invasive proxy for creatine turnover and biodynamics.

